# Reduction ascending aortoplasty: A retrospective analysis of outcomes and risk factors

**DOI:** 10.3389/fcvm.2022.953672

**Published:** 2022-07-25

**Authors:** Philipp Szalkiewicz, Johannes Gökler, Wolfgang Dietl, Marek Ehrlich, Christoph Holzinger, Günther Laufer, Dominik Wiedemann

**Affiliations:** ^1^Clinical Department of Cardiac Surgery, University Department of Surgery, Medical University of Vienna, Vienna, Austria; ^2^Clinical Department of Cardiac Surgery, University Hospital of St. Pölten, St. Pölten, Austria

**Keywords:** reduction ascending aortoplasty, ascending aortic aneurysm repair, aortic aneurysm (thoracic), aorta thoracic, ascending aortic surgery

## Abstract

**Objectives:**

Indication for Reduction of Ascending Aortoplasty (RAA) and long-term outcomes remain unclear. This study analyzed the outcomes after nonreinforced RAA in two Austrian centers.

**Methods:**

Patients with RAA at two Austrian centers between 6/2,009 and 6/2,017 were retrospectively analyzed. Aortic diameters were measured by CT pre- and post-operatively. Patients were assigned according to valve morphology and imaging modality.

**Results:**

Overall, 253 patients underwent RAA [women: 30.8%; median age 74 (63–79) years] with a mean preoperative ascending diameter of 44.7 (±3.5) mm. RAA-related postoperative adverse events occurred in 1.2% (*n* = 3) over a follow-up of a median of 3.8 (2.4–5.5) years: One type A aortic dissection, one lethal aortic rupture at the suture line, and one suture line bleeding with cardiac tamponade and need of surgical revision. The overall survival rate was 89.7%. Aortic valve morphology itself was no risk factor for mortality (Log-Rank: 0.942). One hundred and forty patients had a tricuspid [TAV: (55.3%)] aortic valve and 113 patients had a bicuspid aortic valve [BAV: (44.7%)]. Redilatation to a diameter >50 mm according to CT follow-up occurred in 5.7% (*n* = 5 of 87). One patient needed reoperation with RAA and aortic valve replacement due to a prosthesis-patient mismatch after aortic valve replacement and aortic redilatation.

**Conclusion:**

Non-reinforced RAA is a safe, feasible, and reproducible procedure with low rates of perioperative complications in selected patients primarily undergoing aortic valve repair with a dilated ascending aorta. Aortic valve morphology has no impact on mortality after RAA.

## Introduction

Reduction ascending aortoplasty (RAA) is considered a potential alternative to aortic replacement in patients with borderline ascending aortic aneurysms. It is conducted as a concomitant procedure mainly in aortic valve replacement. However, the indication for RAA is unclear. Aortic diameters are considered a prognostic tool for estimating the risk of aortic dissection, rupture, and overall patient outcome (1, 2). Current guidelines recommend ascending aneurysm replacement at aortic diameters of 55 mm in patients with tricuspid aortic valves (TAV), or 50 mm in patients with bicuspid aortic valves (BAV) in presence of risk factors or in patients with genetic connective tissue disorders (1, 2). Although RAA is not recommended as the first-line procedure, it is suggested applicable in patients of higher age and unfit for extended aortic surgery (1), while connective tissue disorders are clear contraindications (3–5). High preoperative aortic diameters are assumed to promote redilatation (6, 7) and in bicuspid aortic valves (BAV), aortic redilatation after RAA is suspected due to underlying histopathologic aortic wall changes (8). Various surgical RAA techniques exist (9): Non-reinforced RAA has been demonstrated to preserve the Windkessel-Effekt while bearing risks of redilatation (10). Supported RAA by external aortic wrapping with dacron prothesis aims for aortic stabilization (11), with the risk of prothesis dislocation and aortic tissue lesions (12). This study analyzed the outcome after nonreinforced RAA and the risk factors that are associated with worse outcomes.

## Patients and methods

### Study design

This study is a retrospective cohort analysis of patients after nonreinforced RAA. Patient data were collected from the clinical department of cardiac surgery at the Medical University Vienna and from University Hospital St.Pölten between 6/2,009 and 6/2,017, including mortality data with the department's annual data request from the mortality registry of Statistic Austria (STAT)—the Austrian statistical office. Information obtained by telephone follow-up was added in this regard.

### Surgical technique of reduction ascending aortoplasty

**Figure 1:**
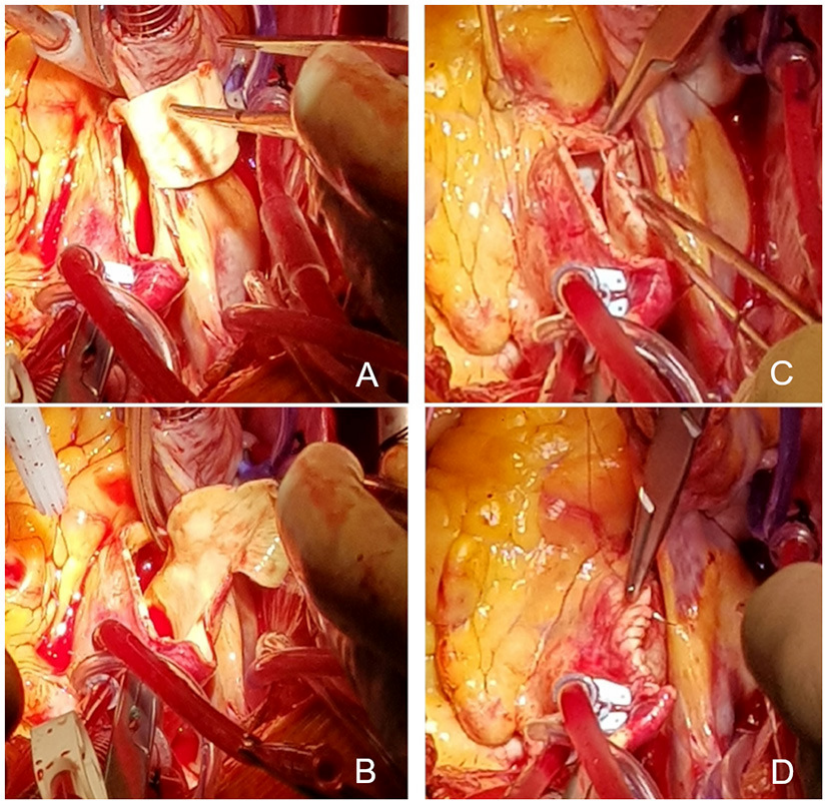
Reduction Ascending Aortoplasty by tissue resection in a wedge-shaped fashion **(A,B)** and suture line completion in a double-layered fashion **(C,D)**.

Non-reinforced RAA was conducted according to the same standardized technique at both hospitals. A wedge-shaped segment of the ascending aorta was excised by performing a longitudinal aortotomy starting next to the aortic clamp at the greater curvature and extending down to the non-coronary sinus (Figure 1). Aortotomy was closed with a continuous 4-0/5-0 prolene suture in a double-layered fashion. Additional suture line augmentation with a pericardial strip for further stabilization was occasionally carried out if considered necessary. Both centers considered RAA feasible in patients with isolated ascending aortic aneurysms with diameters of 40–50 mm, while aortic syndromes such as Marfan-Syndrome are considered a contraindication. RAA was always conducted as a concomitant surgery, mainly in patients with indications for aortic valve surgery. In rare cases of high-risk patients being considered unfit for ascending aortic replacement due to an impaired postprocedural patient outcome being expected, the RAA is chosen as an alternative procedure and thus even exceptionally carried out in patients with ascending aortic diameters exceeding 50 mm.

### Aortic diameter assessment

Aortic diameters were measured preoperatively and during follow-up by CT scan with an angle corrected approach next to the pulmonary artery bifurcation. Aortic diameters exceeding 50 mm during the follow-up were defined as significant redilatation, as those patients were considered to have increased risk for aortic-related complications such as aortic rupture and dissection.

### Statistical analysis

The data of the patients were analyzed using SPSS statistical software version 20 (IBM Corp, Armonk, NY, USA). Continuous variables are shown either as mean and standard deviation (±SD) or median and interquartile range (IQR), categorical as numbers and percentages. For comparison Chi^2^-Test was used, Fisher's exact test for a small sample size. Survival was compared using Kaplan Meyer Analysis and Log-Rank Testing. *P*-values of < 0.05 were defined as statistically significant. Due to inter- and intra-observer variability of aortic diameter assessment, the highest diameter measurement was used in each patient.

## Results

### Patient baseline characteristics and conducted procedures

Patient demographics are depicted in Table 1. Among 253 RAA patients, 78 (30.8%) were women. The median age at the time of surgery was 74 (63–79) years. The median post-operative follow-up was 3.8 (2.4–5.5) years. The primary surgery was predominantly aortic valve replacement (AVR) [*n* = 232 (91.7%)] with aortic stenosis as the primary indication for surgery in 195 (77.1%) patients. Combined aortic vitium was present in 150 (59.3%) patients. The aortic valve was tricuspid in 140 (55.3%) patients and bicuspid in 113 (44.7%). Data on procedural and periprocedural data, including surgical demographics, are given in Table 2. A total of 18 (7.1%) patients had previous cardiac surgery. Then, 3 patients had prior aortic surgery [*n* = 3 (1.2%)]: Two patients had an infrarenal aneurysm replacement and one [*n* = 1 (0.4%)] had a prior reduction ascending aortoplasty. All data analyzed regarding this patient are related to the secondary RAA procedure in the present study. No other patient needed ascending aortic reoperation. Preoperative diameters in CT scans were available in 241 (95.3%) patients. Average diameters were 44.7 mm (±3.5) with maximum diameters of 56 mm. A total of 87 (34.4%) patients had postoperative CT scans with a median imaging follow-up of 16 (2–48) months. Mean values for the highest and last measured diameters according to imaging modalities are depicted in Table 3.

**Table 1:** Patient and surgical baseline characteristics of the study population.

***n*** **(all)**	**253 (100%)**
**Demographics**	
Female, *n* = (%)	78 (30.8)
Height in cm, median (IQR)	174 (166–180)
Weight in kg, mean (±SD)	82.9 (16.3)
BMI, median (95%-IQR)	27.3 (24.7–31.0)
Age in years, median (IQR)	74 (63–79)
AV Stenosis, *n* = (%)	195 (77.1)
Combined aortic vitium	150 (59.3%)
TAV, *n* = (%)	140 (55.3)
BAV, *n* = (%)	113 (44.7)
Sievers classification, *n* = (%)	
Type 1, *n* = (%)	56 (22.1)
Type 2, *n* = (%)	4 (1.6)
Type 0, *n* = (%)	41 (16.2)
Type undocumented, *n* = (%)	12 (4.7)
MV Stenosis, *n* = (%)	8 (3.2)
MV Insufficiency, *n* = (%)	168 (66.4)
TV Insufficiency, *n* = (%)	109 (43.1)
**Comorbidities**	
EuroSCORE II, median (IQR)	4.9 (2.8–8.9)
COPD, *n* = (%)	81 (36.0)
CAD, *n* = (%)	25 (9.9)
Hyperlipidemia, *n* = (%)	162 (64.0)
Hypertension, *n* = (%)	201 (79.4)
Diabetes mellitus II, *n* = (%)	48 (19.0)
Stroke, *n* = (%)	17 (6.7)
Myocardial infarction, *n* = (%)	23 (9.1)
CHD, *n* = (%)	93 (36.8)
Angina pectoris, *n* = (%)	66 (26.1)
Atrial fibrillation, *n* = (%)	48 (19)

AV, Aortic Valve; BAV, Bicuspid Aortic Valve; BMI, Body Mass Index; CAD, Cranial Arterial Disease; CHD, Coronary Heart Disease; COPD, Chronic Obstructive Pulmonary Disease; EuroSCORE II, Updated European System for Cardiac Operative Risk Evaluation; IQR, Interquartile Range; MV, Mitral Valve; SD, Standard Deviation; TAV, Tricuspid Aortic Valve; TV, Tricuspid Valve.

**Table 2:** Procedural and periprocedural characteristics.

***n*** **(all)**	**253 (100%)**
**Periprocedural data**	
Elective procedure, *n* = (%)	225 (82.9)
Minimally invasive upper hemisternotomy approach, *n* = (%)	59 (23.3)
Perfusion time (minutes), median (IQR)	111 (89–154)
Aortic cross clamp time (minutes), median (IQR)	79 (61–105.5)
ICU stay (days), median (IQR)	2 (1–3)
Hospital stay (days), median (IQR)	9 (7–13)
**Cardiac procedure**	
AV surgery, *n* = (%)	235 (92.9)
MV surgery, *n* = (%)	37 (14.6)
TV surgery, *n* = (%)	15 (5.9)
CABG, *n* = (%)	63 (24.9)
**Prior cardiac surgery**	
AV surgery, *n* = (%)	17 (6.7)
Aortic surgery, *n* = (%)	3 (1.2)
Ascending aortic surgery, *n* = (%)	1 (0.4)
**Cardiac and aortic reoperation**	
AV surgery, *n* = (%)	6 (2.4)
Aortic surgery, *n* = (%)	3 (1.2)
Ascending aortic surgery, *n* = (%)	0

Aortic surgery: two patients underwent previous surgical infrarenal aortic aneurysm replacement by dacron prothesis and one patient underwent prior Reduction Ascending Aortoplasty; AV, Aortic Valve; AVR, Aortic Valve Replacement; CABG, Coronary Aortic Bypass; ICU, Intensive Care Unit; IQR, Interquartile Range; MV, Mitral Valve; TV, Tricuspid Valve.

**Table 3:** Aortic diameters before and after reduction ascending aortoplasty, measured in mm by computed tomography.

**Preoperative aortic diameter [*****n*** **=** **241 (100%)]**	
mean (±SD)	44.7 (3.5)
Max. diameter	56
40 ≤ 45, *n* = (%)	105 (43.6)
45 ≤ 50, *n* = (%)	97 (40.2)
50 ≤ 55, *n* = (%)	23 (9.5)
>55, *n* = (%)	1 (0.4)
**Postoperative aortic diameter [*n* = 87 (100%)]**	
Highest, mean (±SD)	40.6 (5.8)
Last, mean (±SD)	40.2 (5.7)
Max. diameter	70
40 ≤ 45, *n* = (%)	32 (36.8)
45 ≤ 50, *n* = (%)	12 (13.8)
>50, *n* = (%)	5 (5.7)

Mean diameter is stated for the highest measured diameters (Highest) and the last (Last) in each patient follow-up.

### Overall patient outcome

**Figure 2:**
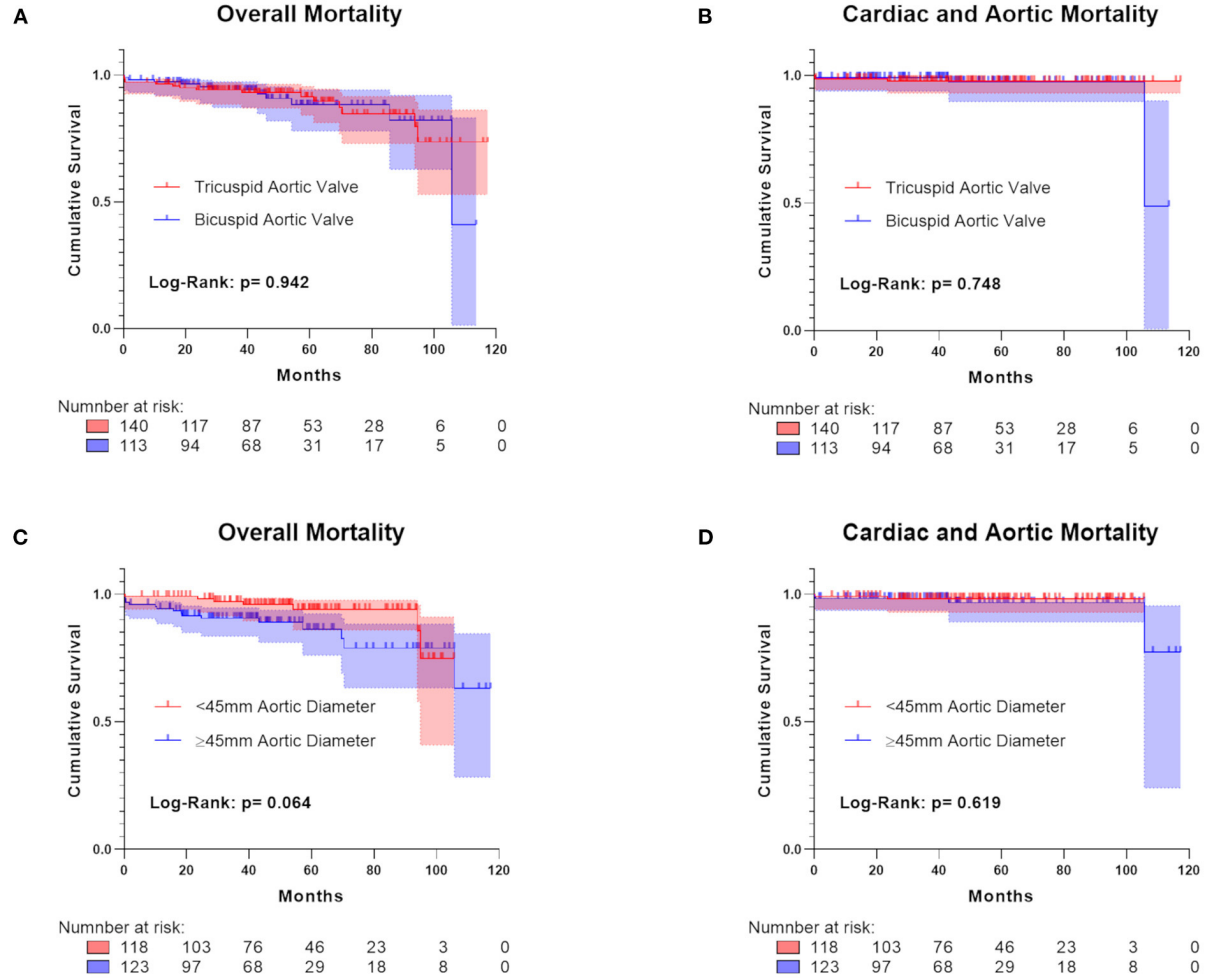
Kaplan-Meier survival analyses regarding overall mortality and combined cardiac and aortic mortality between patients with tricuspid and bicuspid aortic valves **(A,B)**, and between patients with aortic diameters <45 and ≥45 mm **(C,D)**.

The most common complication was atrioventricular block III in 27 (11.6%) patients (see Table 4). Major neurological events (ischemic stroke) occurred in 14 (5.5%) patients, while pericardial tamponade occurred in 4 (1.6%) patients. There was no intraoperative death, and one in-hospital death due to AV-block induced asystole after aortic valve replacement (0.4%). The 30-day mortality was 2% (*n* = 5), including two patients with cardiac causes of death. One patient was the already mentioned AV-Block induced asystole, and the second experienced acute heart failure after hospital discharge. Moreover, one patient died due to an aortic rupture at the aortoplasty suture line within 2 weeks after hospital discharge. This patient with reportedly preoperative aortic diameters of 50.4 mm according to CT imaging represents the only case of RAA-related cause of death. Overall, six (2.4%) patients experienced either cardiac or aortic mortality, with the according survival rate for the combined cardiac and aortic-related cause of death being 97.6% in our study population. Overall mortality during the follow-up was 10.3% (*n* = 26). Kaplan-Meier analyses on mortality outcomes are presented in Figure 2. Patients with different valve morphology did not differ in overall mortality (Log-Rank: 0942) and combined cardiac and aortic mortality (Log-Rank: 0.748). Moreover, there was no significant difference in overall mortality (Log-Rank: 0.064) and combined cardiac and aortic mortality (Log-Rank: 0.619) between patients with ascending aortic diameters <45 and ≥45 mm, which have been measured in 118 (49%) and 123 (51%) patients.

**Table 4:** Postoperative complications.

***n*** **(all)**	**253 (100%)**
Overall mortality, *n* = (%)	26 (10.3)
RAA—related, *n* = (%)	3 (1.2)
Type A dissection, *n* = (%)	1 (0.4)
Aortic rupture, *n* = (%)	1 (0.4)
RAA suture line bleeding	1 (0.4)
Myocardial infarction, *n* = (%)	7 (2.8)
Pericardial tamponade, *n* = (%)	4 (1.6)
Atrial fibrillation, *n* = (%)	63 (24.9)
AV-block III, *n* = (%)	27 (11.6)
Stroke, *n* = (%)	14 (5.5)
Pulmonary embolism, *n* = (%)	5 (2)

AV-Block, Atrioventricular Block; RAA, Reduction Ascending Aortoplasty.

### RAA related complications

Out of 87 available CT follow-ups, five (5.7%) patients had significant redilatation with aortic diameters > 50 mm. Complications related to RAA are depicted in Table 4. A total of three patients experienced RAA-related events [*n* = 3; (1.2%)]: One patient had a prior mentioned lethal aortic rupture at the suture line. The second case was an early postoperative surgical bleeding at the suture line which could be surgically revised. The third adverse event was a Stanford type A dissection, linked to redilatation of the ascending aorta during follow-up, which occurred 79 months after the initial RAA. This patient had a progressive thoraco-abdominal aortic aneurysm with ascending aortic diameters of 70 mm according to CT imaging and contraindication for reoperation due to comorbidities. One patient had an ascending aorta redilatation which was promoted by a poststenotic aortic valve based on a patient prothesis mismatch (PPM). This patient had a repeat AVR and RAA 68.9 months after the initial surgery.

## Discussion

In this retrospective analysis that includes 253 patients with nonreinforced reduction ascending aortoplasty due to ascending aortic aneurysms, the safety and efficacy of this strategy were confirmed. The valve morphology (TAV/BAV) itself does not impact the overall mortality.

Despite skepticism within the surgical community, this study shows that nonreinforced RAA results in excellent short and long-term outcomes. The outcome is comparable to international data on isolated aortic valve replacement (13). The only procedural difference between RAA and aortic valve replacement is the aortotomy across the ascending aorta including aortic tissue resection. This is reflected by the fact that the majority of reported surgical complications are not RAA related but can be associated with the primary surgical intervention.

Early RAA-related complications are limited to aortoplasty suture line bleeding. This is presumably based on exerted tension, stress, and pressure peaks, all increasing the early risk for tearing at the suture line. Noteworthy, the according risk is increased in aortas with rigid and thinned walls (14), predisposing to suture line bleeding and aortic rupture (5, 6). Potential late complications include redilatation and aneurysm progression or dissection. Generally, reoperation and aortic dissection rates are reported extremely seldom (3, 5, 7, 15). Our overall 98.8% freedom of RAA-related complications is comparable to other research (4) and our overall survival of 89.7% and freedom from aortic and cardiac-related death of 97.6% is excellent. In comparison, Hwang et al. report on 10-year survival rates of 91.1% and on 10-year freedom from cardiac-related death of 96.2% for nonreinforced RAA (15).

Aortic wall tension and consecutive risk of rupture are lowered by diameter reduction (16). Impaired aortic wall quality with reduced aortic wall thickness is associated with aortic dilatation (17), rupture, or dissection (16). Therefore, adequate pre- and intraoperative tissue quality assessment and patient selection are crucial. Reported significant redilatation rates >50 mm were low and without clinical relevance in most patients. It remains speculative whether redilatation is more likely to arise in patients with inadequate surgical technique: The resected aortic segment might be of irrelevant size leading to little aortic diameter reduction or the suture line might not be continued across the complete ascending aorta leaving aneurysmatic aorta. Postoperative aortic diameter reduction to at least 35 mm is emphasized (7).

Aortic valve pathologies are considered to have an impact on ascending aortic aneurysm formation (1, 2). However, valve morphology itself appears not to have an overall impact on redilatation according to certain research (7, 15). Although AVR might suppress ascending aortic aneurysm growth in patients with diseased tricuspid and bicuspid aortic valves, the aorta might still progress in diameter growth postoperatively (18). Noteworthy, the initial valve pathology itself, either regurgitation or stenosis, might predispose to differences in postoperative diameter increase after AVR as well (19).

Data on long-term feasibility and risk factors of RAA, as well as on its comparison to aortic replacement are scarce. Carrell et al. compared external supported RAA and ascending aortic replacement, both in patients undergoing AVR, with those receiving valved conduits. Their data reveal superior mortality outcomes and less postoperative bleeding and cerebrovascular complications after RAA (20). Belov et al. compared external supported RAA and ascending aortic replacement in a small patient collective without any further cardiac procedures. Their results emphasize the shorter aortic cross-clamp and cardiopulmonary bypass times, comparable to Carrel et al. (20) and Belov et al. (21). Nevertheless, at that time there are no adequate state-of-the-art data on comparing contemporary outcomes after RAA vs. aortic replacement in patients undergoing AVR. RAA is considered a simpler and less radical technique than aortic replacement (7, 15, 22). As there exist several RAA techniques, there is no adequate data on their comparison. Non-reinforced RAA reportedly maintains the Windkessel-Effect (10). Contrary, reinforced RAA with aortic wrapping as external support aims for aortic stabilization (11) and diameter control (7) but bears the risk of wall lesions from prothesis dislocation (12).

Precise patient follow-up is substantial. As RAA is an aortic surgery, adequately structured follow-up with regular control intervals is needed. The follow-up has to include risk prevention and treatment assessment. Since RAA was a concomitant procedure in all patients, strict follow-up was not executed in all our patients which might expose the patient to the dispensable risk of unrecognized redilatation and missed opportunities for proper treatment evaluation and adaptation. To distinguish between the postoperative residual aneurysm and recurrent aneurysm with redilatation, a CT scan before hospital discharge can provide an accurate reference point for risk evaluation. Regular CT scans for follow-up are relevant for the estimation of redilatation after surgery and should be weighed against the burden of radiation exposure. TTE facilitates routine measurements. Current guidelines do not provide any precise strategy or recommendations for the follow-up after RAA (1, 2) and there exist no data in this regard.

## Limitations

The study's major limitation is its retrospective character, with follow-up imaging conducted not uniformly and in only a small patient count, resulting in potential analysis bias. Noteworthy, inter- and intra-observer variations during diameter assessment are inevitable. Moreover, this study enables evaluation of the RAA only to a limited extent, as an outcome compared to the common aortic replacement technique was not conducted.

## Conclusion

Aortic dilatation <50 mm was managed safely with RAA technique in our patient cohort and can be considered an alternative approach to aortic replacement. We report on very low RAA-related surgical complications and a low risk of aortic redilatation. It is suitable as a concomitant procedure, mainly in patients with indications for aortic valve surgery.

## Data availability statement

The raw data supporting the conclusions of this article will be made available by the authors, without undue reservation.

## Ethics statement

The studies involving human participants were reviewed and approved by the Local Ethics Committee of the Medical University of Vienna and the State's Ethics Committee of Lower Austria. Written informed consent for participation was not required for this study in accordance with the national legislation and the institutional requirements.

## Author contributions

PS: conceptualization, data curation, formal analysis, investigation, methodology, project administration, supervision, validation, visualization, writing—original draft, and writing—review and editing. JG and DW: conceptualization, investigation, methodology, project administration, supervision, validation, and writing—review and editing. WD: conceptualization, project administration, supervision, validation, and writing—review and editing. ME, CH, and GL: conceptualization and writing—review and editing. All authors contributed to the article and approved the submitted version.

## Conflict of interest

The authors declare that the research was conducted in the absence of any commercial or financial relationships that could be construed as a potential conflict of interest.

## Publisher's note

All claims expressed in this article are solely those of the authors and do not necessarily represent those of their affiliated organizations, or those of the publisher, the editors and the reviewers. Any product that may be evaluated in this article, or claim that may be made by its manufacturer, is not guaranteed or endorsed by the publisher.
